# Supplementary material for: Machine learning coarse-grained potentials of protein thermodynamics
Source: Nat Commun. 2023 Sep 15;14:5739. doi: 10.1038/s41467-023-41343-1 (PMC10504246; doi:10.1038/s41467-023-41343-1)
Supplement: Supplementary file 1 — Supplementary Information [file 41467_2023_41343_MOESM1_ESM.pdf]

Supplementary Information
Machine Learning Coarse-Grained Potentials of Protein Thermodynamics

Maciej Majewski,[†,‡,§§] Adrià Pérez,[†,‡,§§] Philipp Thölke,[†] Stefan Doerr,[‡] Nicholas E. Charron,[¶,§,‖] Toni Giorgino,[⊥] Brooke E. Husic,[#,@,△,∇] Cecilia Clementi,[∗,‖,§,¶,††] Frank Noé,[∗,‡‡,#,‖,††] and Gianni De Fabritiis[∗,†,‡,¶¶]

†Computational Science Laboratory, Universitat Pompeu Fabra, Barcelona Biomedical Research Park (PRBB), Carrer Dr. Aiguader 88, 08003, Barcelona, Spain

‡Acellera Labs, Doctor Trueta 183, 08005, Barcelona, Spain

¶Department of Physics, Rice University, Houston, TX 77005, USA

§Center for Theoretical Biological Physics, Rice University, Houston, TX 77005, USA

‖Department of Physics, FU Berlin, Arnimallee 12, 14195 Berlin, Germany

⊥Biophysics Institute, National Research Council (CNR-IBF), 20133 Milan, Italy

#Department of Mathematics and Computer Science, FU Berlin, Arnimallee 12, 14195 Berlin, Germany

@Lewis Sigler Institute for Integrative Genomics, Princeton University, Princeton, NJ 08540, USA

△Princeton Center for Theoretical Science, Princeton University, Princeton, NJ 08540, USA

∇Center for the Physics of Biological Function, Princeton University, Princeton, NJ 08540, USA

††Department of Chemistry, Rice University, Houston, TX 77005, USA

‡‡Microsoft Research AI4Science, Karl-Liebknecht Str. 32, 10178 Berlin, Germany

¶¶Institució Catalana de Recerca i Estudis Avançats (ICREA), Passeig Lluis Companys 23, 08010 Barcelona, Spain

§§Equal contribution

E-mail: cecilia.clementi@fu-berlin.de; franknoe@microsoft.com; gianni.defabritiis@upf.edu

Contents

List of Supplementary Tables **4**

List of Supplementary Figures **4**

List of Supplementary Listings **4**

Supplementary References **22**

List of Supplementary Tables

1 Table S1 5
2 Table S2 7
3 Table S3 9
4 Table S4 16
5 Table S5 17

List of Supplementary Figures

1 Figure S1 6
2 Figure S2 8
3 Figure S3 9
4 Figure S4 10
5 Figure S5 11
6 Figure S6 12
7 Figure S7 12
8 Figure S8 13
9 Figure S9 13
10 Figure S10 14
11 Figure S11 15
12 Figure S12 18
13 Figure S13 19

List of Supplementary Listings

1 Listing S1 20
2 Listing S2 21

Table S1: Sequences of the proteins used for training. (*) In the sequence of Villin, “X” stands for the non-standard amino-acid norleucine (NLE).

Protein	Sequence
Chignolin	YYDPETGTWY
Trp-Cage	DAYAQWLKDGGPSSGRPPPS
BBA	EQYTAKYKGRTFRNEKELRDFIEKFKGR
WW-Domain	KLPPGWEKRMSRSSGRVYYFNHITNASQWERPSG
Villin (*)	LSDEDFKAVFGMTRSAFANLPLWXQQHLXKEKGLF
NTL9	MKVIFLKDVKGMGKKGEIKNVADGYANNFLFKQGLAIEA
BBL	GSQNNDALSPAIRRLLAEWNLDASAIKGTGVGGRLTREDVEKHLAKA
Protein B	LKNAIEDAIAELKKAGITSDFYFNAINKAKTVEEVNALVNEILKAHA
Homeodomain	RPRTAFSSEQLARLKREFNENRYLTERRRQQLSSELGLNEAQIKIWFQNKRAKI
Protein G	DTYKLVIVLNGTTFTYTTEAVDAATAEKVFKQYANDAGVDGEWTYDAATKTFTVTE
α3D	MGSWAEFKQRLAAIKTRLQALGGSEAELAAFEKEIAAFESELQAYKGKGNPEVEALRKEAAAIRDELQAYRHN
λ-repressor	PLTQEQLEDARRLKAIYEKKKNELGLSQESVADKMGMGQSGVGALFNGINALNAYNAALLAKILKVSVEEFSPSIAREIY

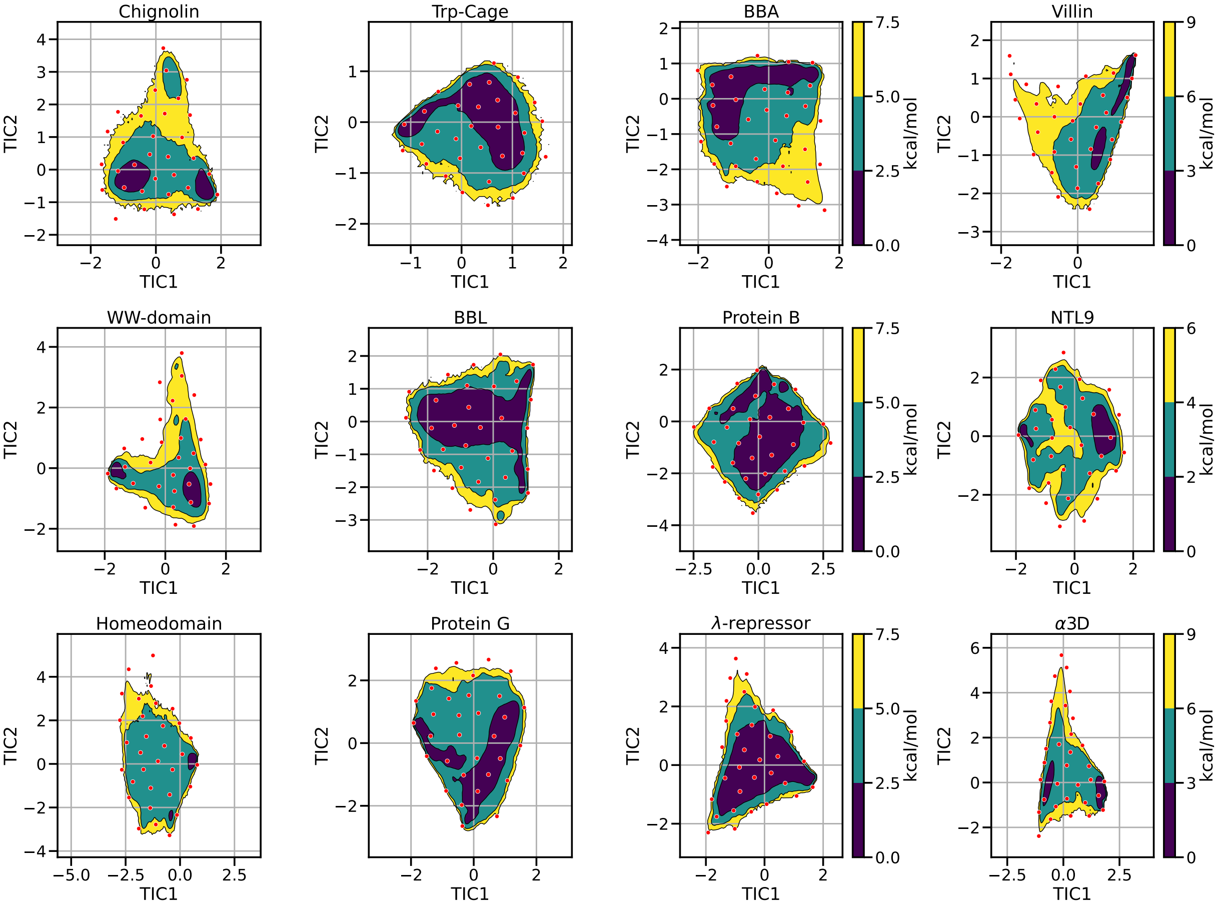

Figure S1: **Overlay of coarse-grained molecular dynamics starting points on all-atom MD free energy surfaces.** Starting points (red dots) of coarse grained molecular dynamics overlayed on top of free energy surface across the first two TICA dimensions for each protein. The free energy surfaces were computed based on the reference all-atom MD. The colorbar shows the energy values in the range from 0 to 9 kcal/mol for Villin and α3D, 6 kcal/mol for NTL9, and 7.5 kcal/mol for the remaining proteins. Source data are provided as a Source Data file.

Table S2: Additional native macrostate statistics from all MSMs built with reference MD simulations and CG simulations from all protein-specific models and the multi-protein model. The data describes different metrics for the identified native macrostate of each protein, showing averages (with standard deviation) and minimum / maximum values for GDT score values, radius of gyration (RG) and fraction of native contacts (FNC)

Protein	Model	Mean GDT	Max GDT	Mean RG (Å)	Min RG (Å)	Mean FNC	Max FNC
	Protein-specific	96 ± 6	100	5.0 ± 0.1	4.6	0.99 ± 0.02	1.00
Chignolin	Multi-protein	87 ± 11	100	5.0 ± 0.1	4.2	0.98 ± 0.03	1.00
	MD Reference	90 ± 8	100	5.0 ± 0.1	4.4	0.98 ± 0.02	1.00
	Protein-specific	65 ± 6	91	6.7 ± 0.2	6.0	0.91 ± 0.03	1.00
Trp-Cage	Multi-protein	64 ± 5	90	6.6 ± 0.2	5.9	0.94 ± 0.03	1.00
	MD Reference	69 ± 11	100	7.1 ± 0.9	6.1	0.94 ± 0.03	1.00
	Protein-specific	54 ± 8	82	7.9 ± 0.4	6.7	0.94 ± 0.03	0.99
BBA	Multi-protein	50 ± 8	85	8.2 ± 0.4	7.1	0.90 ± 0.02	0.99
	MD Reference	53 ± 12	91	11.1 ± 2.5	7.2	0.91 ± 0.04	1.00
	Protein-specific	67 ± 6	92	9.3 ± 0.2	8.2	0.93 ± 0.02	0.99
WW-Domain	Multi-protein	13 ± 5	41	9.7 ± 1.3	7.1	0.62 ± 0.04	0.80
	MD Reference	66 ± 12	98	9.8 ± 1.0	8.2	0.91 ± 0.03	0.99
	Protein-specific	63 ± 10	96	9.2 ± 0.4	8.0	0.92 ± 0.02	0.99
Villin	Multi-protein	59 ± 9	92	8.8 ± 0.3	7.9	0.92 ± 0.02	0.99
	MD Reference	62 ± 21	100	9.8 ± 1.6	8.1	0.88 ± 0.06	1.00
	Protein-specific	72 ± 11	99	9.0 ± 0.4	8.1	0.88 ± 0.03	0.98
NTL9	Multi-protein	24 ± 7	75	10.1 ± 1.2	7.5	0.68 ± 0.05	0.90
	MD Reference	85 ± 13	100	8.6 ± 0.4	8.0	0.92 ± 0.05	1.00
	Protein-specific	46 ± 9	78	10.4 ± 0.6	8.7	0.85 ± 0.02	0.96
BBL	Multi-protein	42 ± 12	77	10.5 ± 0.7	8.7	0.87 ± 0.02	0.96
	MD Reference	46 ± 12	83	10.5 ± 2.2	8.7	0.83 ± 0.04	0.96
	Protein-specific	40 ± 4	69	9.1 ± 0.2	8.2	0.77 ± 0.03	0.88
Protein B	Multi-protein	54 ± 5	73	9.4 ± 0.2	8.5	0.82 ± 0.02	0.89
	MD Reference	48 ± 12	87	11.6 ± 2.5	8.7	0.78 ± 0.04	0.93
	Protein-specific	45 ± 6	67	10.6 ± 0.5	9.5	0.88 ± 0.02	0.95
Homeodomain	Multi-protein	48 ± 6	71	10.9 ± 0.4	9.7	0.88 ± 0.02	0.95
	MD Reference	51 ± 17	100	14.0 ± 3.7	9.6	0.89 ± 0.04	1.00
	Protein-specific	65 ± 5	87	10.6 ± 0.5	9.8	0.92 ± 0.02	0.98
Protein G	Multi-protein	71 ± 5	88	10.6 ± 0.1	10.0	0.94 ± 0.02	0.98
	MD Reference	66 ± 20	100	10.8 ± 0.8	9.7	0.78 ± 0.05	1.00
	Protein-specific	54 ± 2	65	12.6 ± 0.1	12.0	0.85 ± 0.01	0.90
α3D	Multi-protein	54 ± 2	70	12.5 ± 0.2	11.7	0.85 ± 0.01	0.89
	MD Reference	56 ± 7	76	14.8 ± 2.3	11.3	0.89 ± 0.02	0.90
	Protein-specific	40 ± 5	69	12.0 ± 0.2	11.1	0.83 ± 0.02	0.91
λ-repressor	Multi-protein	40 ± 5	65	12.2 ± 0.4	11.3	0.82 ± 0.01	0.88
	MD Reference	50 ± 11	95	13.8 ± 3.1	10.7	0.89 ± 0.04	0.97

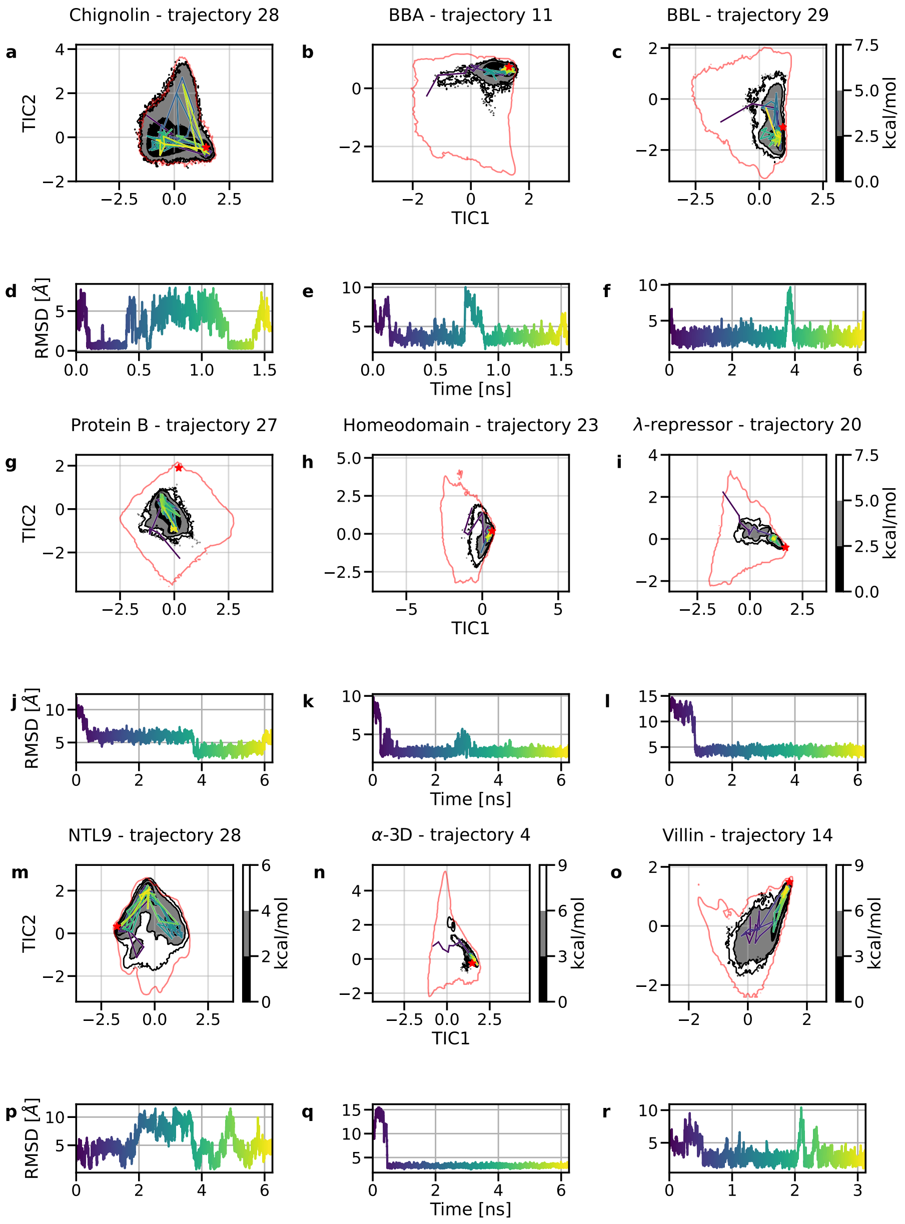

Figure S2: **Selected individual coarse-grained trajectories and α-carbon RMSD for the proteins simulated with protein-pecific models.** Individual CG trajectories selected for 9 of the proteins. Each visualized simulation explores the free energy surface, accesses multiple major basins and shows transitions between native and coil states. On the top panel of each sub-figure: 100 states sampled uniformly from the trajectory plotted over CG free energy surface of (a) Chignolin, (b) BBA, (c) BBL, (g) Protein B, (h) Homeodomain, (i) λ-repressor, (m) NTL9, (n) α3D, (o) Villin. The red line indicates the all-atom equilibrium density by showing the energy level above free energy minimum with the values of 9 kcal/mol for α3D, 6 kcal/mol for NTL9, and 7.5 kcal/mol for the remaining proteins. On the bottom panel: RMSD of α-carbon with the reference to the crystal structure of (d) Chignolin, (e) BBA, (f) BBL, (j) Protein B, (k) Homeodomain, (l) λ-repressor, (p) NTL9, (q) α3D, (r) Villin. The remaining proteins are included in Figure 2. Source data are provided as a Source Data file.

Table S3: Visualizations of representative trajectories, where each protein reaches the native state, generated using coarse-grained simulation with NNP. The selected trajectories correspond to trajectories selected in Figure 2 and Supporting Figure 2. The trajectories for WW-Domain, NTL9, BBL, Protein B and Protein G were split into parts, visualizing transitions between states and separated by 2 s of blank frames. The movies were produced with Open-Source PyMOL[1] with period of 0.5 ps per frame.

Protein	Trajectory	Time frame [ns]	Video URL
Chignolin	28	0.0 - 0.615	https://youtu.be/vs5jf_3VheA
Trp-Cage	16	1.49 - 2.6	https://youtu.be/l9MI6XQZjnU
BBA	11	0.0 - 1.0	https://youtu.be/G9PnPkELl7E
WW-Domain	4	1.1 - 1.6 & 9.5 - 10.0	https://youtu.be/dBDOKvZ4CS4
Villin	14	2.08 - 2.5	https://youtu.be/beQVf8YEpXI
NTL9	28	3.4 - 3.9 & 4.5 - 5.0	https://youtu.be/CSOaCFcx0Ho
BBL	29	0.0 - 0.5 & 3.5 - 4.0	https://youtu.be/lwia6Z6ik9k
Protein B	27	0.0 - 0.5 & 3.5 - 4.0	https://youtu.be/7l5Zavu25eg
Homeodomain	23	0 - 1.0	https://youtu.be/QG0gQJwwvxM
Protein G	14	0.0 - 0.2 & 3.3 - 3.4 & 7.3 - 8.2	https://youtu.be/Ozt63Z9yB3w
α3D	4	0.0 - 0.8	https://youtu.be/56LDlUbptpE
λ-repressor	20	0.0 - 1.1	https://youtu.be/0U10m_MgC7g

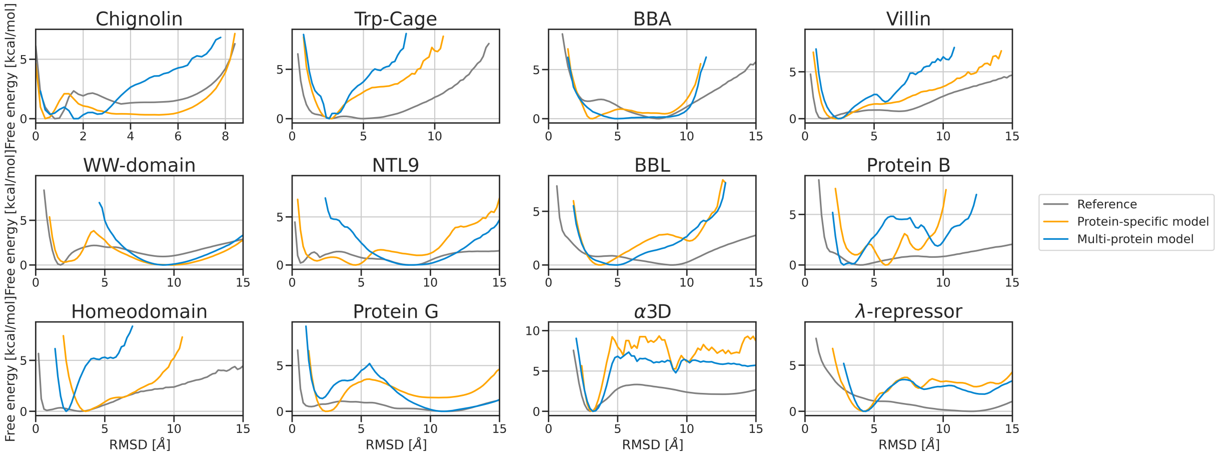

Figure S3: **Comparison of all-atom and coarse-grained free energy plots over Cα-RMSD.** Free energy plot over Cα-RMSD for the all-atom MD and CG models with PDB structure as the reference. The reference PDB is listed on Figure 1. Source data are provided as a Source Data file.

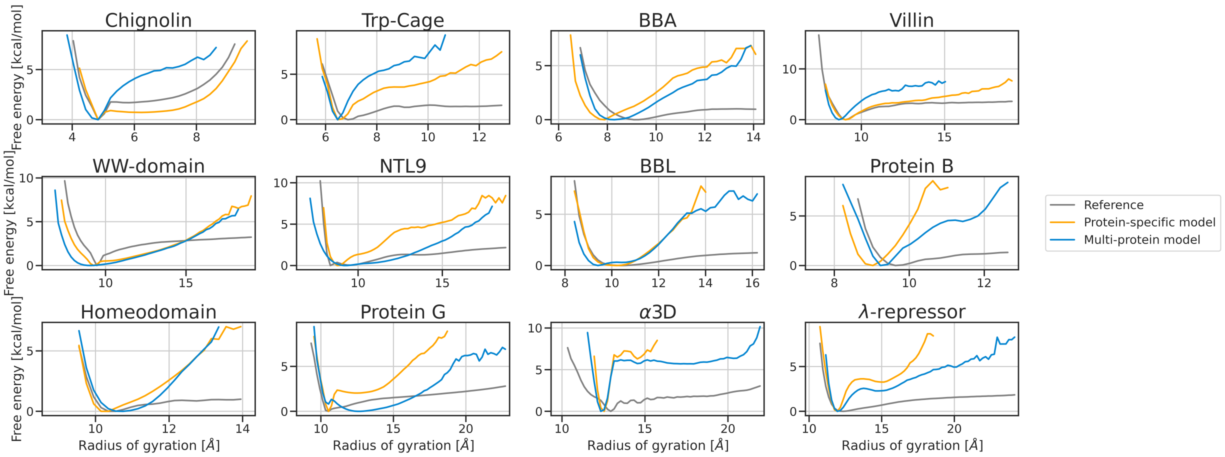

Figure S4: **Comparison of all-atom and coarse-grained free energy plots over radius of gyration.** Free energy plot over radius of gyration for the all-atom MD and CG models, computed only with Cα atoms. Source data are provided as a Source Data file.

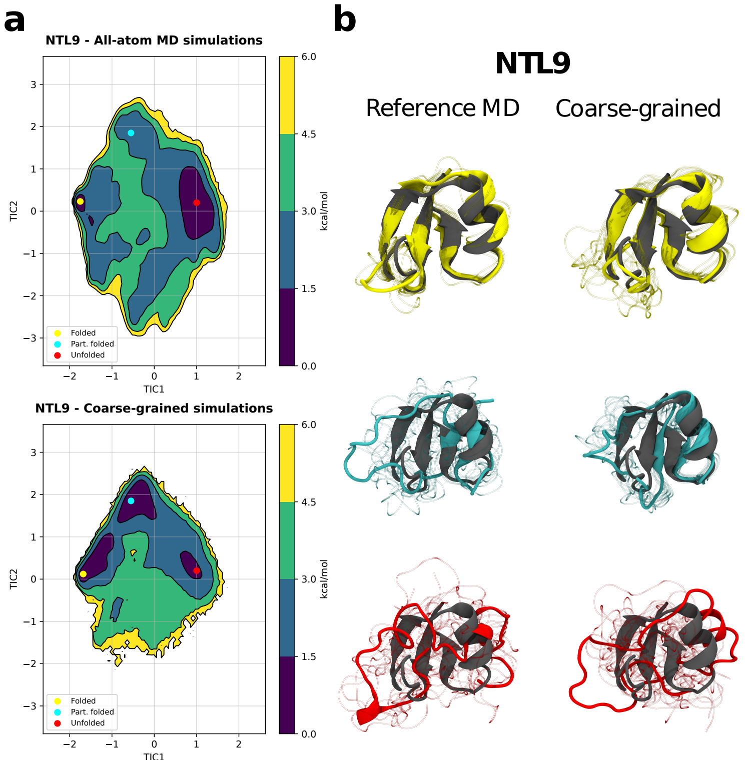

Figure S5: **Comparison of free energy surfaces and sampled conformations for NTL9 using all-atom MD and coarse-grained simulations.** (a) Free energy surface of NTL9 over the first two TICs for the all-atom MD simulations (top) and the coarse-grained simulations (bottom) using the protein-specific model. The circles identify different relevant minima. (b) Sampled conformations from the macrostates corresponding to the marked minimas in the free energy surfaces for NTL9. On the left column, conformations from the all-atom MD simulations are shown. On the right column, conformations sampled from the coarse-grained simulations are shown. Sampled structure colors correspond to the minima colors in the free energy surface plot, with blurry lines of the same color showing additional conformations from the same state. Reference experimental structures for NTL9 (PDB: 2HBA) are colored in gray. Source data are provided as a Source Data file.

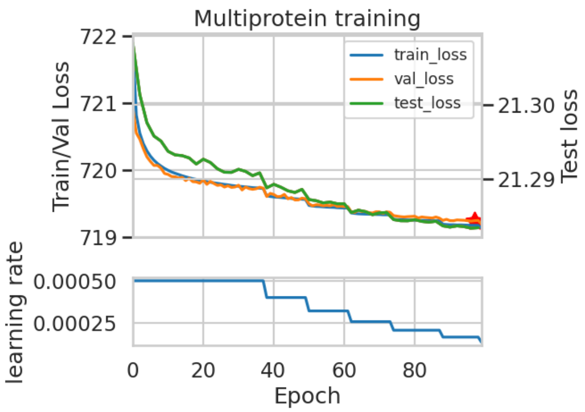

Figure S6: **Training curves for the multi-protein model trained on all the proteins.** Top panel: training (blue, *train_loss*), validation (orange, *val_loss*) and test loss (green, *test_loss*). The reported metrics are L1 loss for the training and validation loss and MSE loss for the test loss. Bottom panel: learning rate values across the training of the corresponding models. The model selected is marked with a red star. Source data are provided as a Source Data file.

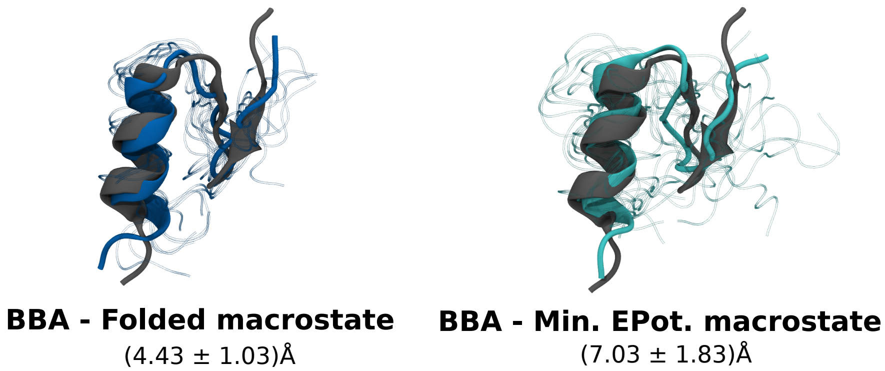

Figure S7: **Sampled structures from minimum RMSD and potential energy macrostates in coarse-grained simulations of BBA.** Sampled structures of BBA obtained from CG simulations of the multi-protein model, coming from the minimum RMSD macrostate (left, deep blue) and the minimum potential energy macrostate (right, cyan). In gray, the reference experimental structure of BBA (PDB: 1FME) for comparison. Structures were sampled from each respective macrostate. Ten conformations were sampled from each conformational state (visualized as transparent shadows)and the lowest RMSD conformation of the sampled structures is displayed in cartoon representation, reconstructing the backbone structure from α-carbon atoms. Text indicates the protein name, the corresponding macrostate from where structures where sampled, and mean RMSD and standard deviation of such macrostates.

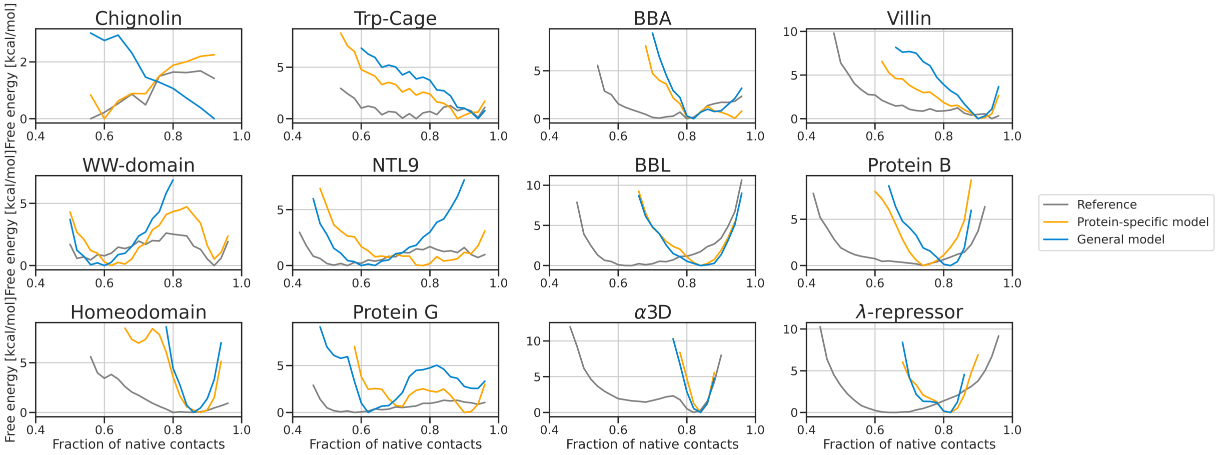

Figure S8: **Comparison of all-atom and coarse-grained free energy plots over fraction of native contacts.** Free energy plot over fraction of native Cα contacts for the all-atom MD and CG models. Reference PDB used for the native contacts is listed on Figure 1. Source data are provided as a Source Data file.

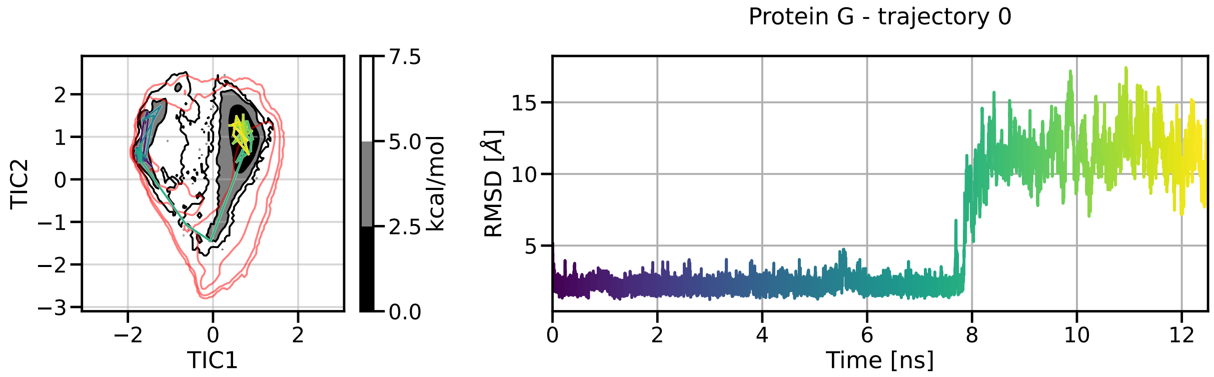

Figure S9: **Selected representative coarse-grained trajectory of Protein G simulated with the multi-protein model.** The visualized simulation explores the free energy surface, accesses multiple major basins and shows transitions between native and coil states. On the left panel: 100 states sampled uniformly from the trajectory plotted over CG free energy surface. The red line indicates the all-atom equilibrium density by showing the energy level above free energy minimum with the value of 7.5 kcal/mol. On the right panel: RMSD of α-carbon with the reference to the crystal structure. The remaining trajectories are available in the GitHub repository associated with this publication. Source data are provided as a Source Data file.

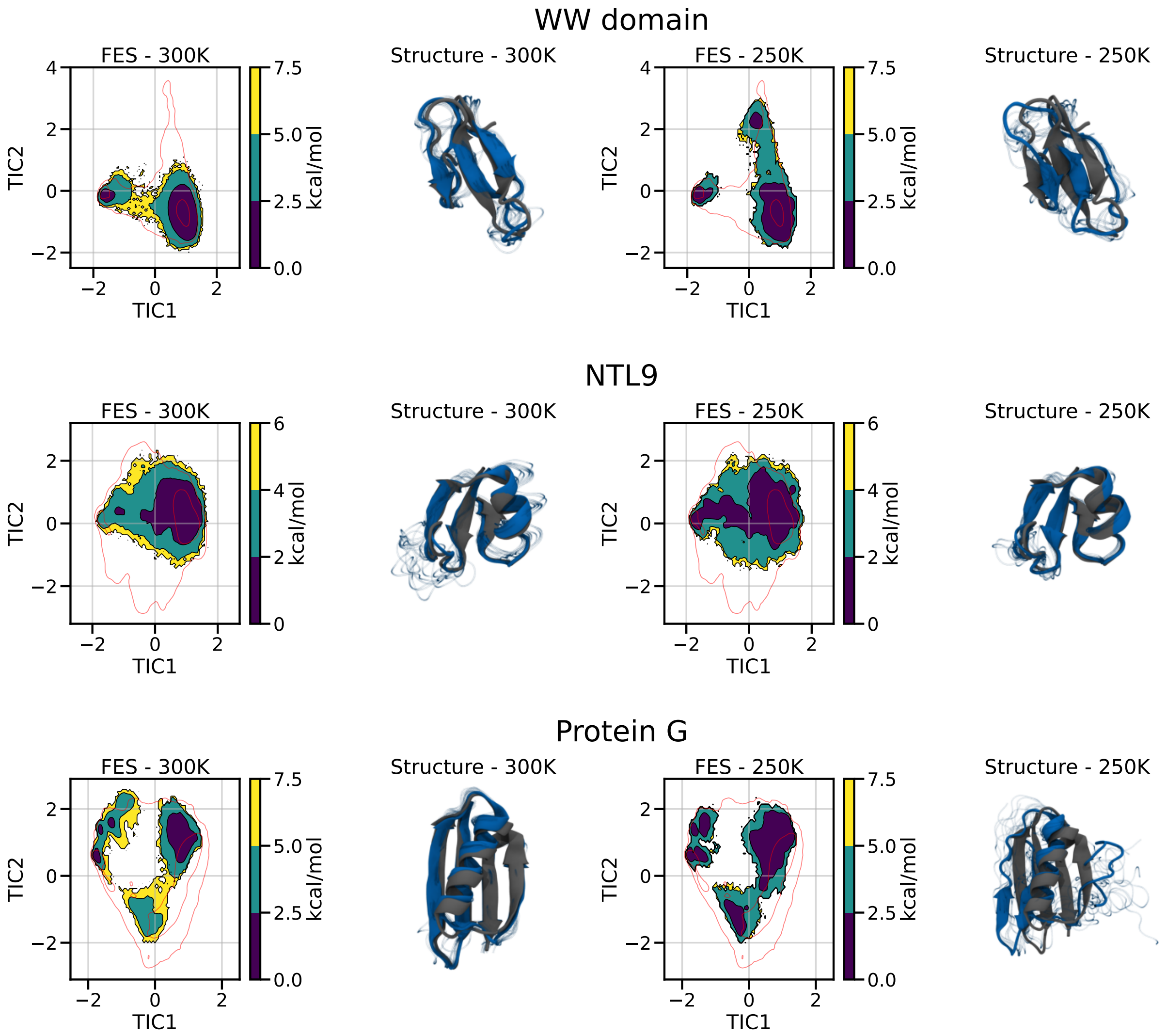

Figure S10: **Results of coarse-grained simulation for WW domain, NTL9, and Protein G at lower temperatures.** Results of CG simulation with the multi-protein model at lower temperatures (300K and 250K) for WW domain, NTL9 and Protein G. The results for each protein are presented in individual row from left to right: Free energy surface at 300K; Representative structure of the native macrostate at 300K; Free energy surface at 250K; Representative structure of the native macrostate at 250K. The red line indicates the all-atom equilibrium density by showing the energy level above free energy minimum with the values of 6 kcal/mol for NTL9 and 7.5 for WW-domain and Protein G. Source data are provided as a Source Data file.

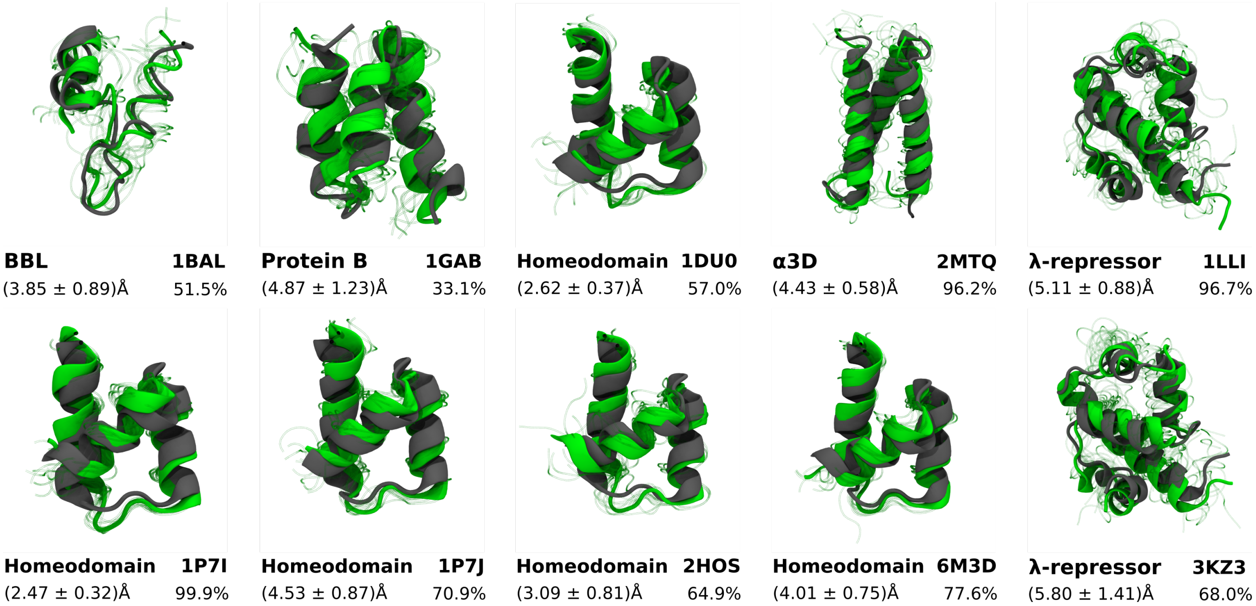

Figure S 11: **Representative structures for selected mutants retrived from the CG simulations made from the multi-protein model.** Structures were sampled from the native macrostate, which was identified as the macrostate containing the conformation with the minimum RMSD with the reference to the mutant's crystal structure. Ten conformations were sampled from the native macrostate, and the backbone was reconstructed for the most similar one to the crystal structure, for visual purposes. In grey, the reference crystal structure. In green, the sampled structure with the backbone reconstructed. The green blurry structures are the remaining structures sampled from the same macrostate. Text indicates protein name and PDB ID. Values on the left indicate the average RMSD of the native macrostate, and percentage shows its equilibrium probability.

Table S4: Summary of selected protein mutants. The mutants that kept the native structure in a preliminary validation are presented in Table 3. The structures of recovered native macrostates of mutants that sampled transition to netive state are shown in Supporting Figure 11. Amino-acid substitutions in the sequences are bolded, insertions are underlined and deletions are marked as ’-’.

Protein	PDB	# mutations	Kept native structure	Transition to native state	Sequence
Chignolin	1UAO	2	No	No	**G**YDPETGTW**G**
Trp-Cage	1L2Y	3	No	No	**NL**Y**I**QWLKDGGPSSGRPPPS
BBA	1FSD	2	No	No	**Q**QYTAK**I**KGRTFRNEKELRDFIEKFKGR
BBA	1PSV	8	No	No	**KP**YTA**RI**KGRTF**S**NEKELRDF**L**E**T**F**T**GR
NTL9	1CQU	1	No	No	MKVIFLKDVKG**K**GKKGEIKNVADGYANNFLFKQGLAIEA
Protein B	1GAB	2	Yes	Yes	LKNA**K**EDAIAELKKAGITSDFYFNAINKAKTVEEVNAL**K**NEILKAHA
Protein B	2N35	10	Yes	No	LK**E**A**K**E**K**AI**E**ELKKAGITSD**Y**YF**DL**INKAKTVE**G**VNAL**KD**EILKAHA
BBL	1BAL	3	Yes	Yes	**EE**QNNDALSPAIRRLLAE**H**NLDASAIKGTGVGGRLTREDVEKHLAKA
Homeodomain	1DU0	1	Yes	Yes	RPRTAFSSEQLARLKREFNENRYLTERRRQQLSSELGLNEAQIKIWF**A**NKRAKI
Homeodomain	1P7I	1	Yes	Yes	RPRTAFSSEQLARLKREFNENRYLTERRRQQLSSELGLNEAQIKIWFQN**A**RAKI
Homeodomain	1P7J	1	Yes	Yes	RPRTAFSSEQLARLKREFNENRYLTERRRQQLSSELGLNEAQIKIWFQN**E**RAKI
Homeodomain	2HOS	4	Yes	Yes	RPRTAFSSEQLARLKREFNENRYLTERRRQQLSSELGLNEAQ**V**K**G**WF**K**N**M**RAKI
Homeodomain	6M3D	2	Yes	Yes	RPRTAFSSEQLARLKREFNENRYLTERRRQQLSSELGLNEAQIKIWF**K**NK**A**AKI
Protein G	1GB1	10	No	No	**M**TYKL- - **I**LNG**K**T**L**KG**ET**TTEAVDAATAEKVFKQYAND**N**GV<br>DGEWTYD**D**ATKTFTVTE
Protein G	2GI9	11	No	No	**MQ**YKL- - **I**LNG**K**T**L**KG**ET**TTEAVDAATAEKVFKQYAND**N**GV<br>DGEWTYD**D**ATKTFTVTE
Protein G	2J52	9	No	No	**M**TYKL- - **I**LNG**K**T**L**KG**ET**TTEAVDAATAEKVFKQYAND**N**GV<br>DGEWTYDAATKTFTVTE
Protein G	5BMG	11	No	No	**MQ**YKL- - **I**LNG**K**T**L**KG**CT**TTEAVDAATAEKVFKQYAND**N**GV<br>DGEWTYD**D**ATKTFTVTE
Protein G	1EM7	15	No	No	**T**TYKL- - **I**LNG**K**T**L**KG**ET**TTEAVDA**E**TAE**R**VFK**E**YA**KKN**GV<br>DGEWTYD**D**ATKTFTVTE
Protein G	1FCL	15	No	No	**TTF**KL**I**I- - NG**K**T**L**KG**ET**TTEAVDAATAEKV**L**KQY**I**ND**N**G**I**<br>DGEWTYD**D**ATKT**W**TVTE
Protein G	1MPE	16	No	No	**MQ**YK- - **VI**LNG**K**T**L**KG**ET**TTEAVDAAT**F**EKV**V**KQ**FF**ND**N**GV<br>DGEWTYD**D**ATKTFTVTE
Protein G	1P7E	14	No	No	**MQ**YKLVI- - NG**K**T**L**KG**ET**TT**K**AVDA**E**TAEK**A**FKQYAND**N**GV<br>DG**V**WTYD**D**ATKTFTVTE
Protein G	1Q10	15	No	No	**MQ**YK- - **VI**LNG**K**T**L**KG**ET**TTEAVDAATAEKV**V**KQ**FF**ND**N**GV<br>DGEWTYD**D**ATKTFTVTE
Protein G	2KLK	12	No	No	**MQ**YKL- - **I**LNG**K**T**L**KG**ET**TTEAVDAATAEKVFKQY**F**ND**N**GV<br>DGEWTYD**D**ATKTFTVTE
Protein G	2ON8	16	No	No	**MQF**KL**I**I- - NG**K**T**L**KG**EI**T**L**EAVDAA**E**AEK**K**FKQYAND**N**G**I**<br>DGEWTYD**D**ATKTFTVTE
Protein G	2ONQ	16	No	No	**MQF**KL**I**I- - NG**K**T**L**KG**EI**T**I**EAVDAA**E**AEK**F**FKQYAND**N**G**I**<br>DGEWTYD**D**ATKTFTVTE
Protein G	3V3X	13	No	No	**MQ**YKL**IL**CGK**T**L**K**G**E**T- - - TTEAVDAATAE**C**VFKQYAND**N**GV<br>DGEWTYD**D**ATKTFTVTE
Protein G	4WH4	14	No	No	**MQ**YKL**HL**HGK**T**L**K**G**E**T- - - TTEAVDAATAE**H**VFK**H**YAND**N**GV<br>DGEWTYD**D**ATKTFTVTE
Protein G	5BMH	12	No	No	**MQ**YKL- - **I**LNG**K**T**L**KG**ET**TTEAVDAATAEKVFKQYAND**N**GV<br>DGEW**C**YD**D**ATKTFTVTE
Protein G	5UB0	13	No	No	**M**T**F**KL**I**I- - NG**K**T**L**KG**ET**TTEAVDAATAEKV**L**KQYAND**N**G**I**<br>DGEWTYD**D**ATKTFTVTE
Protein G	5UBS	14	No	No	**M**T**F**KL**I**I- - NG**K**T**L**KG**ET**TTEAVDAATAEKVFKQY**F**ND**N**G**I**<br>DGEWTYD**D**ATKTFT**I**TE
Protein G	5UCE	14	No	No	**M**T**F**K**AI**I- - NG**K**T**L**KG**ET**TTEAVDAATAEKVFKQY**F**ND**N**G**L**<br>DGEWTYD**D**ATKTFTVTE
Protein G	6NJF	13	No	No	**M**T**F**KL**I**I- - NG**K**T**L**KG**ET**TTEAVDAATAEKVFKQYAND**N**G**L**<br>DGEWTYD**D**ATKTFT**I**TE
Protein G	6NL8	12	No	No	**M**TYKL- - **I**LNG**K**T**H**KG**EL**TTEAVDAATAEK**H**FKQ**H**AND**L**GV<br>DGEWTYD**D**ATKTFTVTE
Protein G	6NLA	13	No	No	**M**TYKL- - **I**LNG**K**T**H**KG**VL**T**I**EAVDAATAEK**H**FKQ**H**AND**L**GV<br>DGEWTYD**D**ATKTFTVTE
Protein G	3FIL	12	No	No	**MQ**YKL- - **I**LNG**K**T**L**KG**VL**T**I**EAVDAATAEKVFKQYAND**L**GV<br>DGEWTYD**D**ATKTFTVTE
α3D	2MTQ	3	Yes	Yes	MGSWAEFKQRLAAIKTR**C**QALGGSEAE**C**AAFEKE<br>IAAFESELQAYKGKGNPEVEALRKEAAAIRDE**C**QAYRHN
λ-repressor	3KZ3	6	Yes	Yes	**S**LTQEQLEDARRLKAI**W**EKKKNELGLS**Y**ESVADKMGMGQS<br>**A**V**A**ALFNGINALNAYNAALLAKILKVSVEEFSPSIAREI**R**
λ-repressor	1LLI	3	Yes	Yes	PLTQEQLEDARRLKAIYEKKKNELGLSQES**L**ADK**L**GMGQS<br>G**I**GALFNGINALNAYNAALLAKILKVSVEEFSPSIAREIY

Table S5: Hyperparameter choices for the NNP training. The values selected for the final model are bolded. The rest of the paramters available in TorchMD-Net, that are not listed here, were left at default values. The number of RBF was listed as a range, as we tested a large number of different values, ultimately selecting 18.

Hyperparameter	Name	Values tested
Number of interaction layers	num_layers	[1,2,3,**4**,5,6]
Activation function	activation	[**tanh**, ssp]
Radial base function (RBF) type	rbf_type	[gauss, **expnorm**]
Number of RBF	num_rbf	[2-150], **18***
Upper cutoff for RBF	cutoff_upper	[4.0, 6.0, 9.0, **12.0**, 15.0]
Lower cutoff for RBF	cutoff_lower	[0.0, **3.0**, 3.6, 4.1]
Trainable RBF	trainable_rbf	[**true**, false]
Model type	model	graph-network
Embedding dimension	embedding_dimension	[**128**, 256]
Early stopping patience	early_stopping_patience	30
Initial learning rate (LR)	lr	0.0005
LR factor	lr_factor	0.8
Minimal value of LR	lr_min	1.0e-06
LR patience	lr_patience	10
Number of LR warm up steps	lr_warmup_steps	0
Neighbor embedding	neighbor_embedding	false
Saving interval	save_interval	2
Testing interval	test_interval	2
Test set ratio	test_ratio	0.1
Validation set ratio	val_ratio	0.05
Weight decay	weight_decay	[**0.0**, 0.1, 0.001]
Force Terms	-	**Bonds**, Angles, **Dihedrals**, **RepulsionCG**

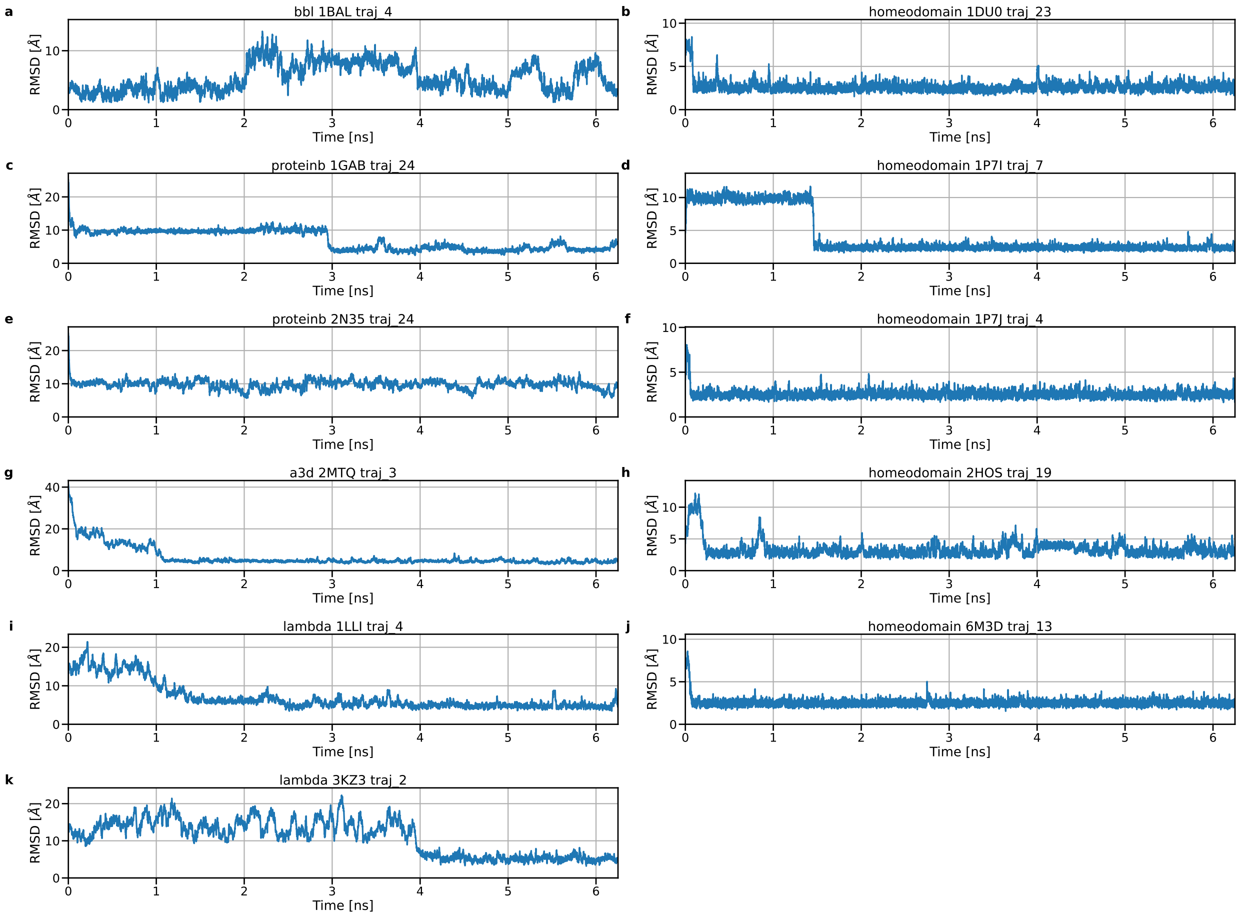

Figure S12: **α-carbon RMSD of selected individual coarse-grained trajectories of mutants.** Individual CG trajectories selected for 11 of the mutants with the RMSD of α-carbon with the reference to the crystal structure (PDBid listed in the title of each subplot) for: (a) BBL mutant 1BAL, (b) Homeodomain - 1DU0, (c) Protein B - 1GAB, (d) Homeodomain - 1P7I, (e) Protein B - 2N35, (f) Homeodomain - 1P7J, (g) α3D - 2MTQ, (h) Homeodomain - 2HOS, (i) λ-repressor - 1LLI, (j) Homeodomain - 6M3D, (k) λ-repressor - 3KZ3. All trajectories are available in the GitHub repository associated with the publication. Source data are provided as a Source Data file.

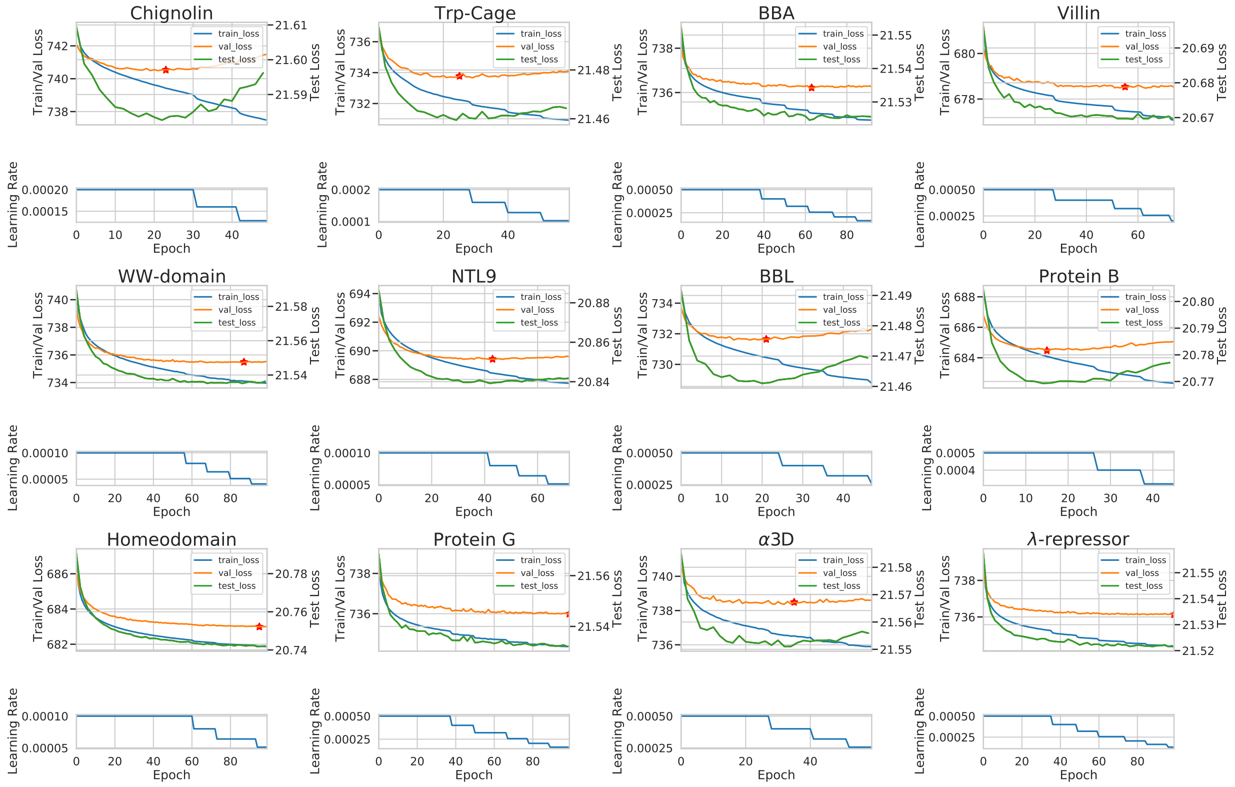

Figure S13: **Training curves for the models trained on individual proteins.** Top panel: training (blue, *train_loss*), validation (orange, *val_loss*) and test loss (green, *test_loss*). The reported metrics are L1 loss for the training and validation loss and MSE loss for the test loss. Bottom panel: learning rate values across the training of the corresponding models. The models selected are marked with a red star. Source data are provided as a Source Data file.

Listing 1: Input file with a set of hyperparameters used for training of the multi-protein model. The protein-specific networks were trained with the same set of hyperparameters, with the exception of the fields “coord_files”, “embed_files” and “force_files”, which were set to the files appropriate for each protein target, and “num_epochs”, which was set to 200.

```yaml
activation: tanh
batch_size: 256
inference_batchsize: 256
dataset: Custom
coord_files: "data/*coords*.npy"
embed_files: "data/*embeddings.npy"
force_files: "data/*deltaforces*.npy"
cutoff_upper: 12.0
cutoff_lower: 3.0
derivative: true
distributed_backend: ddp
early_stopping_patience: 30
embedding_dimension: 128
label:
- forces
lr: 0.0005
lr_factor: 0.8
lr_min: 1.0e-06
lr_patience: 10
lr_warmup_steps: 0
model: graph-network
neighbor_embedding: false
ngpus: -1
num_epochs: 100
num_layers: 4
num_nodes: 1
num_rbf: 18
num_workers: 8
rbf_type: expnorm
save_interval: 2
seed: 94572
test_interval: 2
test_ratio: 0.1
trainable_rbf: true
val_ratio: 0.05
weight_decay: 0.0
```

Listing 2: An example of simulation input file.

```yaml
forcefield: ca_priors-dihedrals_general.yaml
forceterms:
- Bonds
- RepulsionCG
- Dihedrals
exclusions: ('bonds')
langevin_gamma: 1
langevin_temperature: 350
log_dir: cln_32trajs_350_ts1
output: output
output_period: 100
precision: double
replicas: 32
rfa: false
save_period: 1000
seed: 1
steps: 5000000
topology: cln.psf
coordinates: cln_kcenters_32clusters_coords.xtc
temperature: 350
timestep: 1
external:
  module: torchmdnet.calculators.torchmdcalc
  embeddings: [4, 4, 5, 8, 6, 13, 2, 13, 7, 4]
  file: model.ckpt
```

Supplementary References

(1) Schrödinger, LLC, The PyMOL Molecular Graphics System, Version 2.5.0, Schrödinger, LLC. https://github.com/schrodinger/pymol-open-source, 2022.
