# Supplementary material for: Machine learning coarse-grained potentials of protein thermodynamics
Source: Nat Commun. 2023 Sep 15;14:5739. doi: 10.1038/s41467-023-41343-1 (PMC10504246; doi:10.1038/s41467-023-41343-1)
Supplement: Supplementary file 3 — Reporting Summary [file 41467_2023_41343_MOESM3_ESM.pdf]

nature portfolio

Corresponding author(s): Gianni de Fabritiis

Last updated by author(s): Jul 17, 2023

Reporting Summary

Nature Portfolio wishes to improve the reproducibility of the work that we publish. This form provides structure for consistency and transparency in reporting. For further information on Nature Portfolio policies, see our Editorial Policies and the Editorial Policy Checklist.

Statistics

For all statistical analyses, confirm that the following items are present in the figure legend, table legend, main text, or Methods section.

n/a	Confirmed	
☒	☐	The exact sample size (*n*) for each experimental group/condition, given as a discrete number and unit of measurement
☒	☐	A statement on whether measurements were taken from distinct samples or whether the same sample was measured repeatedly
☒	☐	The statistical test(s) used AND whether they are one- or two-sided<br>*Only common tests should be described solely by name; describe more complex techniques in the Methods section.*
☒	☐	A description of all covariates tested
☒	☐	A description of any assumptions or corrections, such as tests of normality and adjustment for multiple comparisons
☐	☒	A full description of the statistical parameters including central tendency (e.g. means) or other basic estimates (e.g. regression coefficient) AND variation (e.g. standard deviation) or associated estimates of uncertainty (e.g. confidence intervals)
☒	☐	For null hypothesis testing, the test statistic (e.g. *F*, *t*, *r*) with confidence intervals, effect sizes, degrees of freedom and *P* value noted<br>*Give P values as exact values whenever suitable.*
☒	☐	For Bayesian analysis, information on the choice of priors and Markov chain Monte Carlo settings
☒	☐	For hierarchical and complex designs, identification of the appropriate level for tests and full reporting of outcomes
☒	☐	Estimates of effect sizes (e.g. Cohen's *d*, Pearson's *r*), indicating how they were calculated

Our web collection on statistics for biologists contains articles on many of the points above.

Software and code

Policy information about availability of computer code

Data collection	All codes are free and available in github.com/torchmd. The code to run molecular dynamics is available at github.com/torchmd/torchmd. The neural network architecture is available at github.com/torchmd/torchmd-net.
Data analysis	The tutorials to reproduce this work are available at github.com/torchmd/torchmd-protein-thermodynamics

For manuscripts utilizing custom algorithms or software that are central to the research but not yet described in published literature, software must be made available to editors and reviewers. We strongly encourage code deposition in a community repository (e.g. GitHub). See the Nature Portfolio guidelines for submitting code & software for further information.

Data

Policy information about availability of data

All manuscripts must include a data availability statement. This statement should provide the following information, where applicable:

- Accession codes, unique identifiers, or web links for publicly available datasets
- A description of any restrictions on data availability
- For clinical datasets or third party data, please ensure that the statement adheres to our policy

The models and data generated in this study are available at \url{github.com/torchmd/torchmd-protein-thermodynamics}. Starting structures for molecular dynamics were sourced from Protein Data Bank \url{https://www.rcsb.org/}, with PDBids: 5AWL, 2JOF, 1FME, 2F4K, 1PIN, 2HBA, 2WXC, 1PRB, 1ENH, 1MI0, 2A3D, 1LMB, 1BAL, 1GAB, 2N35, 1DU0, 1P7I, 1P7J, 2HOS, 6M3D, 2MTQ, 1LLI, 3KZ3. Source data are provided with this paper.

Research involving human participants, their data, or biological material

Policy information about studies with human participants or human data. See also policy information about sex, gender (identity/presentation), and sexual orientation and race, ethnicity and racism.

Reporting on sex and gender	not aplicable
Reporting on race, ethnicity, or other socially relevant groupings	not aplicable
Population characteristics	not aplicable
Recruitment	not aplicable
Ethics oversight	not aplicable

Note that full information on the approval of the study protocol must also be provided in the manuscript.

Field-specific reporting

Please select the one below that is the best fit for your research. If you are not sure, read the appropriate sections before making your selection.

☒ Life sciences ☐ Behavioural & social sciences ☐ Ecological, evolutionary & environmental sciences

For a reference copy of the document with all sections, see nature.com/documents/nr-reporting-summary-flat.pdf

Life sciences study design

All studies must disclose on these points even when the disclosure is negative.

Sample size	The number of systems studied in this work was selected based on previous examples from the literature. We aimed to compare our results to the well-established study of small protein folding [Lindorff-Larsen, Kresten, et al. Science 334.6055 (2011): 517-520.]. The length of all-atom molecular dynamics simulations for neural network potential training was selected based on our knowledge gathered during the previous study [Husic, Brooke E., et al. The Journal of chemical physics 153.19 (2020)] in particular we aimed to sample multiple transitions between conformational states of each protein.
Data exclusions	To avoid biasing the results towards starting conformations, 10% of the initial frames of each trajectory were excluded from the analysis.
Replication	The training of each model was replicated 2 to 4 times with different random seeds. Each replica was then tested by performing a fast validation simulation. All models produced stable molecular dynamics simulations, however, their performance in terms of replication of free energy surface varied. Then, the best-performing replica was selected for the more extensive validation, where each final model was simulated in 32 replicas starting from different initial conformations.
Randomization	Not relevant to the study as it is a purely computational study
Blinding	Blinding was not possible as it is a purely computational study

Reporting for specific materials, systems and methods

We require information from authors about some types of materials, experimental systems and methods used in many studies. Here, indicate whether each material, system or method listed is relevant to your study. If you are not sure if a list item applies to your research, read the appropriate section before selecting a response.

Materials & experimental systems

n/a	Involved in the study	
☒	☐	Antibodies
☒	☐	Eukaryotic cell lines
☒	☐	Palaeontology and archaeology
☒	☐	Animals and other organisms
☒	☐	Clinical data
☒	☐	Dual use research of concern
☒	☐	Plants

Methods

n/a	Involved in the study	
☒	☐	ChIP-seq
☒	☐	Flow cytometry
☒	☐	MRI-based neuroimaging
